# Pilot implementation of elder-friendly care practices in acute care setting: a mixed methods study

**DOI:** 10.1186/s12913-020-05091-y

**Published:** 2020-04-24

**Authors:** Mubashir Aslam Arain, Laura Graham, Armghan Ahmad, Mollie Cole

**Affiliations:** 1 Alberta Health Services, Health Systems Evaluation and Evidence, Provincial Clinical Excellence, 10301 Southport Lane SW, Calgary, AB T2W 1S7 Canada; 2 Alberta Health Services, Seniors Health Strategic Clinical Networks, Calgary, AB Canada

**Keywords:** Elder-friendly care, Acute care, Senior programs, Quality of care, Mixed methodology, Evaluation

## Abstract

**Background:**

Frail older patients are at risk of experiencing a decline in physical and cognitive function unrelated to the reason for admission. The Elder-Friendly Care (EFC) program was designed to improve the care, experiences, and outcomes of frail older adults. The project supported 8 Early Adoption Sites (EAS) in a large Canadian healthcare organization by providing multiple strategies, educational opportunities, and resources. The purpose of this study was to assess the usefulness of EFC educational materials and resources, staff practice changes and perceptions in pilot sites, and readiness for scale and spread.

**Methods:**

The study was conducted from May 2017 to June 2018 using a mixed-methods approach incorporating the Kirkpatrick Model of Training/Evaluation. A total of 76 Direct Care Staff participated in the staff survey, which assessed their awareness of, satisfaction with, and utilization of EFC principles, resources, and practices. Additionally, 12 interviews were conducted with staff who were directly involved in site implementation of EFC.

**Results:**

Most survey participants were aware (86%, *n* = 63) of the EFC program, and 85% (*n* = 41) indicated they or their site/unit had implemented EFC. Out of these 41 participants, the most common practice changes identified were: incorporating alternatives to restraint (81%, *n* = 33), decreased use of pharmacological restraint (78%, *n* = 32), and patient and family care planning (76%, *n* = 31). Participants that attended all 3 EFC Learning Workshops (LWs) were significantly more likely to recommend the EFC Toolkit to others (87% versus 40%; χ^2^ = 8.82, *p* < 0.01) compared to participants attending less than 3 EFC LWs. Interview participants indicated that the program was well structured and flexible as sites/units could adopt changes that suited their individual sites, needs, contexts, and challenges.

**Conclusions:**

The educational materials and resources used for the EFC project are useful and appreciated by the Direct Care Staff. Further, participants perceive the EFC intervention as effective in creating positive practice change and useful in reducing hospital-related complications for older patients. Future implementation will investigate the impact of EFC on system-level outcomes in acute care.

## Background

The world is aging rapidly, and the population of older people is increasing at an unprecedented rate [1]. In Canada, it is estimated that the population of adults aged 65 years or older (hereafter referred to as older adults) will roughly double to 10.47 million by 2036 [2]. Currently, this population accounts for 35% of hospital discharges and 45% of hospital stays in Canada [3]. In 2017, the total health expenditure for older adults was $242 billion, representing 11.5% of Canada’s Gross Domestic Product (GDP) [4]. With this combination of population aging and increased demand for healthcare services leading to higher healthcare spending-to-GDP ratios [5], new principles and practices of care need to be explored and adopted.

During hospital stays, older adults are at risk of experiencing declines in physical and cognitive function unrelated to the reason for admission leading to extended stays, higher care needs at discharge, and emergency department visits and re-admissions after discharge [6]. Around 15% of acute care discharges over age 80 are readmitted within 30 days [7]. In patients discharged to non-home settings, the number of discharge medications and polypharmacy predicted re-hospitalization [8]. Older people taking five or more medications are at higher risk of delirium and falls, independent of medication indications [9]. Falls can lead to physical restraint use and deconditioning, extended Length of Stay (LOS), and result in higher levels of care requirements on discharge [10]. The level of patient and family engagement in care planning for older patients is also unclear, especially when patients have cognitive impairment from delirium or early dementia. Lastly, routine practices such as the use of indwelling catheters and washable under-pads may lead to hospital-acquired wounds and infections [11].

Current care practices for frail older patients in acute care settings require particular attention to reduce the risk in this population [1, 3]. Physical frailty is defined as a medical syndrome with multiple risk factors leading to a decline in physiologic function, resulting in increased vulnerability and higher dependency demands [12]. The scientific literature suggests that frailty perhaps could be reversible if intervened at the appropriate time as the frailty characteristics are discrete from disease and disability [12]. Hence, age-related innovative programs provide an enormous opportunity to enhance care for frail older citizens [11]. Enhanced care in the hospital should set priorities for a strategic approach to care that optimizes the wellbeing and function for older adults. Maintaining a pre-admission level of function for frail older patients by preventing adverse delirium results in improved quality of life and greater satisfaction [13]. The literature suggests the utilization of nursing interventions focused on nutrition, hydration, mobility, comfort, falls prevention, and social support are key practice elements when caring for older patients [14]. The potential health system benefits of reduced post-discharge care needs, restraint use, readmission rates, and LOS when possible will help to lower expenditures costs [10, 15].

A number of studies have argued that elder-friendly hospital system should emphasize the “4 Ms”: matters most, mobility, medications, and mentation [5, 16]. Care should focus on what “matters most” to patients and families, following their care goals and personal needs, while mobility emphasizes keeping older adults active to combat the harmful effects of restraints and immobility in hospitals [16]. Ensuring medications are prescribed carefully and appropriately with the smallest necessary amount and consideration to harmful and unexpected effects can support patient treatment goals, quality of life, and outcomes [16]. Finally, mentation should focus on the cognitive needs and changes of frail older adults, including the role of delirium in acute stays and frail older adults [16]. Most of these programs target nurses and Direct Care Staff to adopt new strategies and changes by providing staff with information and resources for patient care planning and discharge [17]. Educational training regarding intervention principles improves staff practices, decision making, and judgment leading to new processes promoting health gains and preventing harm [18]. These new care processes need to be adopted at the organizational level to achieve results and seek benefits.

### Project details and characteristics

The Elder-Friendly Care (EFC) in Acute Care project is an age-related initiative to improve the care, experiences, and outcomes of frail older adults. The project was developed and implemented by the Alberta Health Services (AHS)^1^ Seniors Health Strategic Clinical Network (SCN),^2^ and supports improvements in care practices through educational opportunities and resources. The project is based on many of the elder-friendly principles and strategies described above, including the “4 M” criteria based on matters most, mobility, medications and mentation [16]. The program fits the “4 M” criteria for EFC: an emphasis on Patient and Family Centred Care (PFCC) supports what “matters most” to patients and families; “mobility” is highlighted throughout educational opportunities and resources; “medication” reviews and reduced pharmacologic restraints are key components of the EFC approach to care; and resources for delirium prevention, responsive behaviours, sleep support, and meaningful activities address “mentation” [5, 16]. Additionally, EFC encourages decreased use of a broad range of restraints,^3^ focuses on identifying and supporting frail older adults,^4^ and stresses PFCC care planning and transitions for successful discharges.

The EFC program tries to achieve the following six goals for frail older patients: 1) Minimize decline in cognitive and physical function 2) Improve patient and family satisfaction, 3) Prevent adverse events such as falls and delirium, 4) Reduce use of pharmacological and mechanical restraints, 5) Reduce the need for increased care service on discharge, and where possible, length of stay, and 6) Address preventable causes of acute care readmissions from the community and continuing care.

The EFC project team adopted the Innovation Learning Collaborative (ILC) approach developed by SCNs in AHS. ILCs “engage provincial teams in using measurement to drive clinical pathway practice changes to achieve system-wide improvements.” ILC methods include learning sessions, action periods, measurement, results and sustainability processes. The ILC method, tools, and training are standardized to achieve: 1) frontline engagement; 2) focus on quality and 3) finish to sustainment. Additionally, the team adopted the PROSCI methodology for change management, which focuses on building awareness, desire, knowledge, ability, and reinforcement of change. For the EFC project, the team developed an online Toolkit, a variety of resources (e.g. tools, posters), and three Learning Workshops (LWs) which were delivered to 8 Pilot sites across the province. These sites were called Early Adoption Sites (EAS); 4 sites were in rural areas and 4 sites in Edmonton. The urban sites in Edmonton implemented the project in 3–6 units rather than the entire hospital due to their larger size. For each EAS, units chose 3–5 staff members responsible for attending workshops, educating, implementing EFC principles and practices, liaising with the EFC project team, collecting data, and reporting progress. These participants were known as EFC Change Team members.

### Description of workshops

The content of the workshops was decided to align with EFC program goals to care for frail older patients. A total of three workshops were offered; 1) Restraint as a Last Resort, 2) Elder Friendly Environment - Support of Sleep and Delirium and 3) Effective Transitions of Frail Patients. The details and focus of each workshop are highlighted below.

Learning Workshop #1: This Workshop introduces the focus of EFC: vulnerability of cognitively and physically frail older adults, and strategies to avoid unintended consequences for patients, system, and staff. Frail patients are frequently restrained, which leads to many unintended consequences. A new Restraint as a Last Resort provincial policy requires significant practice change, and the EFC Project supports this by addressing restraint risks, benefits, and alternative strategies. Care teams are introduced to frailty, behaviour mapping, care planning, medication review, and resources available on the EFC and AUA Toolkits.

Learning Workshop #2: This workshop focuses on the physiology of sleep and why cognitively and physically frail adults are at risk of delirium. Many persons with dementia have trouble sleeping, and common interventions often worsen insomnia and contribute to daytime sedation, night time waking, falls, and confusion. Sleep interruptions and deprivation are especially common in hospitals, due to the decibel levels of common hospital activities. Factors that contribute to delirium include medications, dehydration, malnutrition, infection, stress, and surgery. Antipsychotics and mechanical restraint have commonly been used to “treat” delirium but these can actually cause and/or worsen it. Alternate strategies to support sleep and prevent, identify, and treat delirium are shared with care teams.

Learning Workshop #3: This workshop introduces two AHS approved frailty scales: Edmonton Frail Scale and Clinical Frailty Scale. Strategies are discussed to prevent unintended consequences during and following care in hospitals. These included the mobility/MOVE Project, Patient and Family-Centered Care (PFCC), and support of safe, effective transitions within hospital units and upon discharge to the community.

### Aim and objectives of the study

This study aimed to assess the early development and implementation of EFC principles and practices in 8 acute care EAS within Alberta. The study considers project development, implementation, and staff perceptions of EFC from a process perspective, with emphasis on the effectiveness of educational materials and resources in supporting EAS practice change and project readiness for scale and spread.

## Methods

The study was conducted in a large healthcare organization in Western Canada from May 2017 to June 2018. The study used a mixed methods approach with both quantitative and qualitative methods, using surveys and interviews for data collection. The mixed method approach incorporated the Kirkpatrick Model of Training/Evaluation to assess the impact of EFC educational materials and resources. The details of methods and model are discussed below.

### Kirkpatrick model

The Kirkpatrick model utilizes four levels of outcomes based on reactions (level 1), learning (level 2), behaviour (level 3) and results (level 4). The model determines participants’ reactions based on the degree of favorability to the learning event. Learning is dependent on an individual’s intentions to acquire knowledge, skills, and attitudes. Behaviour is the applicability of training on job tasks and participant’s job role enhancement. Results are obtained as a targeted outcome due to learning events and subsequent reinforcement.

The mixed methods were triangulated to explore six key areas that reflected different levels of the Kirkpatrick’s model. The six key areas were: 1) Use of EFC educational materials; 2) reactions to EFC educational materials; 3) learning from EFC educational materials; 4) transfer, behaviour change and uptake of EFC principles in practice; 5) perceptions of project outcomes; and 6) readiness for Provincial Spread.

Reactions (key area #2) and Learning (key area #3) explored whether EFC materials were effective on Levels 1 and 2 of the Kirkpatrick model by creating positive reactions and supporting staff learning needs. Uptake of EFC principles (key area #4) established whether EFC resources supported Level 3 of effective training, with changes in practice in EAS. Key areas #5 and #6 focused on perceptions of project outcomes and readiness for Provincial Spread, as opposed to the measurement of system-level indicators for project outcomes generally recommended for level 4 of the Kirkpatrick model.

### Direct care staff survey

We defined Direct Care Staff members as workers directly involved in delivering patient care (e.g. RNs, HCAs, PT/OT, pharmacists). The study used a convenience sample identified by EFC site champions and EFC Change Team members to target frontline staff. A survey was used to measure Direct Care Staff awareness of, satisfaction with, and utilization of EFC principles, resources, and practices. The survey consisted of closed and open-ended questions on participant’s use of, satisfaction with, and learning from EFC resources; application of EFC principles into practice; perception of project outcomes; and suggestions for improvement.

Surveys included paper and online versions. Select Survey software was used for the online survey and paper survey packages included instructions and return postage. Both online and paper surveys were distributed approximately 2 months after the majority of EAS had completed the last workshops, in order to give respondents time to make practice changes.

### Interviews

Semi-structured in-depth interviews were conducted with 12 EFC Change Team members using an interview guide developed by authors (see Additional file 1). Interviews were conducted until data saturation was reached and lasted for the duration of 20–30 min.

### Data analysis

The quantitative data collected from the survey were analyzed using descriptive and inferential statistics in Excel 2013 and SPSS 19. Two sets of statistical analyses were conducted: one comparing EFC Change Team member responses to non-members, and the other comparing participants who had attended all 3 workshops to those who attended less than 3. Independent sample t-test and chi-square tests were used at a 95% confidence level. Interviews were anonymized, transcribed, and then coded for emerging themes using Nvivo 11. Coding followed a general inductive approach in which team members conducted multiple close-readings of the transcripts, identified recurring themes and patterns, coded sections of text accordingly, and identified relationships between themes and explanations for findings where possible. The study research questions were used to guide theme development during close-reading and coding [19].

## Results

A total of 76 Direct Care Staff from 8 EAS participated in the survey. 12 EFC Change Team members participated in qualitative interviews.

### Direct care staff survey

A variety of health care practitioners participated in the EFC Direct Care survey; majority were RNs (25%, *n* = 19), LPNs (24%, *n* = 18), Management Staff (12%, *n* = 9), HCA (8%, *n* = 6), Unit Clerks (7%, *n* = 5), others (20%, *n* = 15) and data missing for 5% (*n* = 4). From the 76 direct care staff, only 24% (*n* = 18) attended all three workshops and 26% (*n* = 20) attended none. Eighty-three percent (*n* = 63) of survey participants had heard about the EFC Project, and 45% (*n* = 34) were members of the EFC Change Team at their EAS**.**

#### The EFC toolkit

Out of the 58 participants that responded to the question, around 33% of participants had accessed the EFC Toolkit (*n* = 19) and 36% had thought about doing so (*n* = 21). The remainder did not know what the Toolkit was (22%, *n* = 13) or had not thought about accessing it (9%, *n* = 5).

Around 24 participants reported barriers for not accessing the toolkit. The largest barriers to accessing the Toolkit were poor awareness (46%, *n* = 11) and insufficient time (38%, *n* = 9). Barriers in the “other” category largely consisted of the same challenges with different phrasings and the inability to access the Toolkit because it was online. These findings indicate that the Toolkit is likely under-accessed due to awareness and context-related barriers, though the majority of participants knew it existed and were interested in using it.

**Fig. 1:**
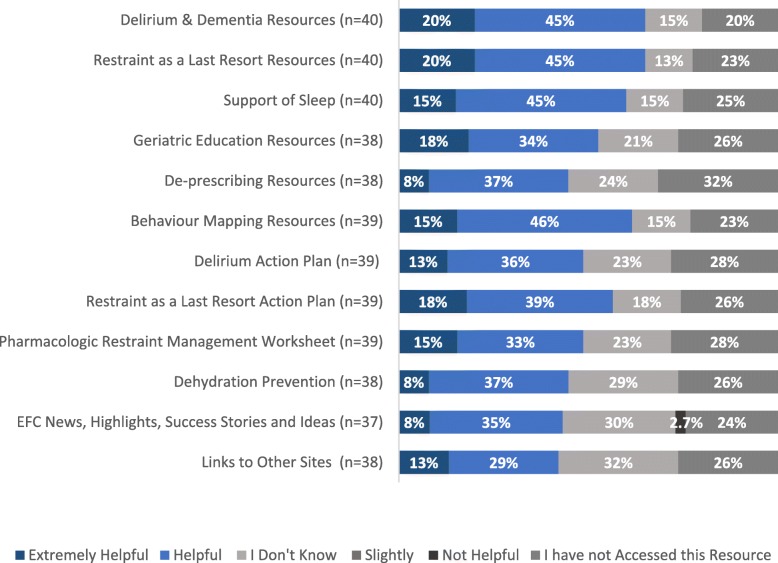
Participant Ratings of EFC Toolkit Resources Helpfulness

Participants reported accessing multiple strategies, tools, and resources available in the Toolkit, although some were utilized more frequently than others. The majority of participants who had accessed the Toolkit found it helpful and identified which resources were most useful (Fig. 1), with delirium and dementia resources and restraint as a last resort ranked the highest.

#### Awareness, learning and practice change

**Fig. 2:**
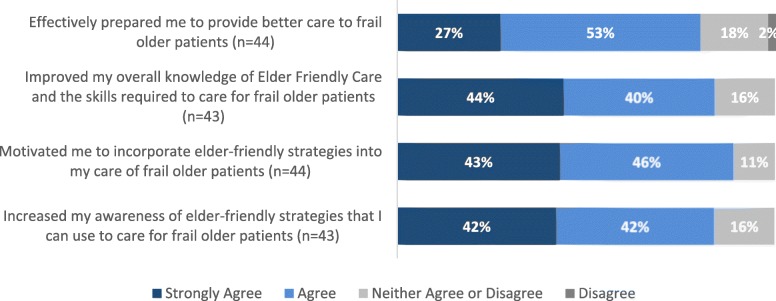
EFC Learning Workshop and Resource Impact on Staff Knowledge, Motivation, Awareness, and Preparedness

A large proportion of participants strongly agreed or agreed with statements regarding the positive impact of workshops and resources on their awareness (84%; 36/43), knowledge (84%; 36/43), preparedness (80%; 35/44) and motivation (89%; 39/44) to adopt EFC (Fig. 2). Around 85% (*n* = 41) of participants also reported that they or their unit/site had implemented EFC strategies to change their approaches to caring for frail older patients.

**Fig. 3:**
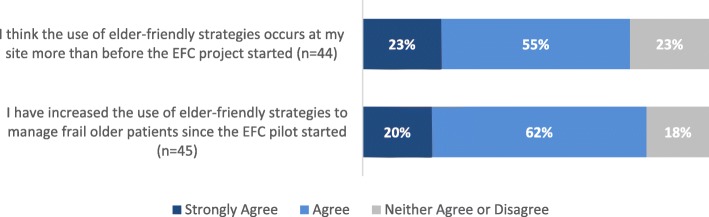
Practice Change at the Site and Individual Levels

**Fig. 4:**
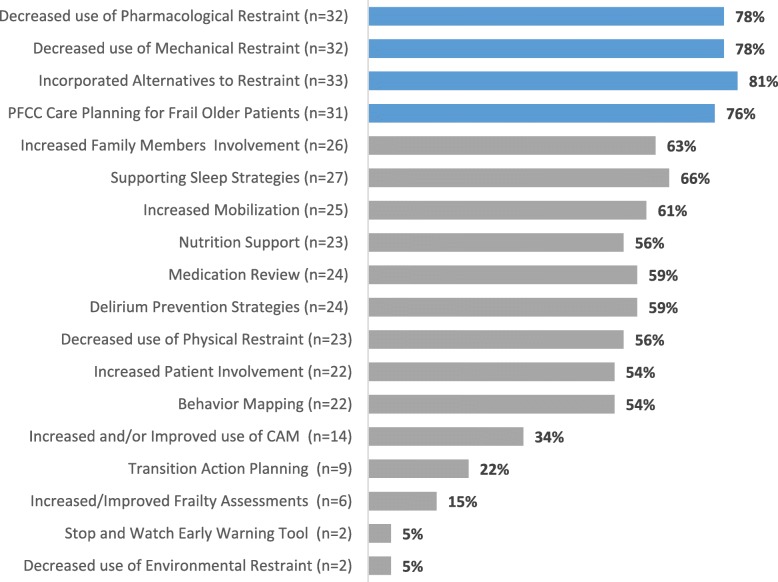
Strategies, Tools, or Resources used by Participants or their Unit

Additionally, the majority agreed or strongly agreed that they had increased their use of elder-friendly strategies since the start of the pilot (82%, 37/45), had their site/unit (78%, 34/44); the remainder were uncertain, but none disagreed (Fig. 3). There was a 78 % (32/41) decrease in pharmacologic and mechanical restraints (e.g. antipsychotics, sedative/hypnotics; lap belts, Broda chairs) because of EFC adopted changes. Also, incorporating alternatives to restraint (81%, 33/41) (e.g. involve the patient, address unmet needs), PFCC care planning (76%, 31/41), and increased family involvement (63%, 26/41) were the other most commonly reported changes as a result of EFC strategies and resources (Fig. 4).

#### Perception of program outcomes

**Fig. 5:**
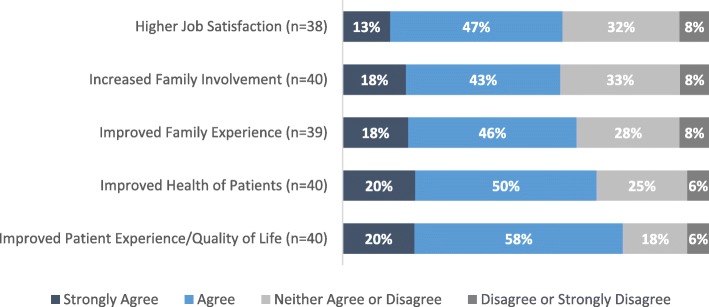
Participant’s Perception of EFC Outcomes

**Fig. 6:**
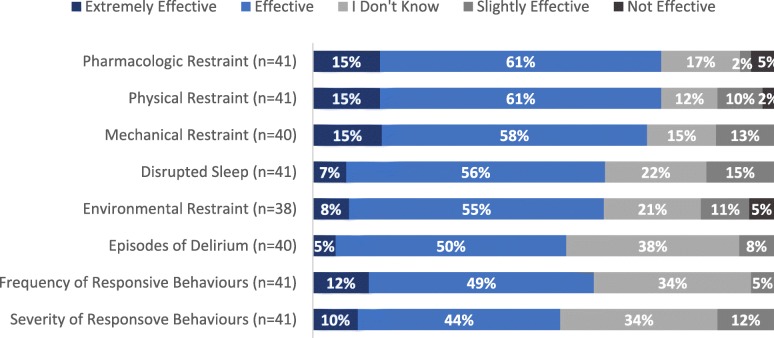
Percent of Survey Participants Reported Effectiveness with EFC in Reducing Targeted Outcomes

Participants agreed or strongly agreed that the EFC project had improved patient experience/quality of life (78%, 31/40), patient health (70%, 28/40), family experience (64%, 25/39) and family involvement (60%, 24/40) (Fig. 5). Participants also rated EFC’s impact on specific domains of care and outcomes covered in EFC LWs (Fig. 6). The majority felt EFC was effective in reducing restraint use, sleep disruption, delirium, and responsive behaviours.

The responses differed significantly between EFC change team members and non-members. Participants that were members of EFC Change Teams were significantly more likely to recommend the EFC Toolkit to others in comparison to non-members (70% versus 29%; χ^2^ = 7.06, *p* < 0.05). No significant differences were found between EFC members and non-members on aspects of utilizing EFC strategies to change the approach to caring for frail older patients (Table 1). Many participants provided ‘don’t know’ responses. There was no statistically significant difference between the characteristic of participants reporting “Don’t Know” responses versus other responses on any question (see Additional file 2).

**Table 1:** Comparison between EFC membership and utilizing EFC resources

Questions	Member of EFC Change Team n (%)	Non-member of EFC Change Team n (%)	χ^**2**^ Statistic	***p***-value
Have you accessed the online EFC Toolkit?				
Yes	14 (41.2%)	4 (17.4%)	8.59	< 0.01*
No	17 (50.0%)	9 (39.1%)		
I don’t Know	3 (8.8%)	10 (43.5%)		
Would you return and use the EFC Toolkit?				
Yes	19 (70.4%)	8 (44.4%)	4.29	0.04*
No	1 (3.7%)	0 (0.00%)		
I don’t Know	7 (25.9%)	10 (55.6%)		
Would you recommend the EFC Toolkit to others?				
Yes	19 (70.4%)	5 (29.4%)	7.06**	0.01*
No	0 (0.00%)	0 (0.00%)		
I don’t Know	8 (29.6%)	12 (70.6%)		
Have you or your unit used EFC strategies to change your approach to caring for frail older patients				
Yes	28 (93.3%)	12 (70.6%)	< 0.01	0.95
No	0 (0.00%)	2 (11.8%)		
I don’t Know	2 (6.70%)	3 (17.6%)		

*Significant differences at 95% confidence level. **Pearson Chi-square test was used for comparison of this question as the ‘No’ responses were zero. For other questions, Chi-Square test for trend was used

Table 2 shows the differences between EFC Change Team members and non-members helpfulness, agreement, and effectiveness scores regarding the EFC Toolkit resources, practice change, and impact on site processes and outcomes, respectively. The members rated higher agreement scores that the EFC toolkit, education, training, or resources improved knowledge and skills (*p*-value = 0.04), effectively prepared participants to provide better care for frail older adults (*p*-value = 0.02), and use of elder-friendly strategies at their site more than before as a result of EFC project (*p*-value = 0.01). EFC members also rated higher for increase in job satisfaction compared to non-members (*p*-value = 0.02), and the effectiveness of the EFC project in reducing disrupted sleep (*p*-value = 0.03) and episodes of delirium (*p*-value = 0.03).

**Table 2:** Comparison of EFC membership regarding resources, practice change, site processes and outcome

Questions	Member of EFC Change Team	Non-member of EFC Change Team	Test Statistic	***p***-value
	Mean (SD)	Mean (SD)		
How helpful are the following EFC Toolkit resources in helping you improve the care you provide to frail older patients?^**a**^				
De-prescribing resources	*n* = 16 4.00 (0.6)	*n* = 10 3.40 (0.5)	2.52	0.02
Links to other sites	*n* = 15 4.07 (0.8)	*n* = 12 3.42 (0.5)	2.44	0.02
Rate your level of agreement with the following statements: **The EFC Toolkit, education, training, or resources I accessed …**^**b**^				
Improved my overall knowledge of Elder Friendly Care and the skills required to care for frail older patients	*n* = 28 4.43 (0.7)	*n* = 14 3.93 (0.7)	0.50	0.04
Effectively prepared me to provide better care to frail older patients	*n* = 28 4.21 (0.7)	*n* = 15 3.67 (0.7)	2.45	0.02
Rate your level of agreement with the following statements: **As a result of the Elder-Friendly care Project...**^**b**^				
I think the use of elder-friendly strategies occurs at my site more than before the EFC project started	*n* = 30 4.20 (0.7)	*n* = 13 3.61 (0.5)	2.83	0.01
Rate your level of agreement with the following statements: **On my unit as a result of the Elder Friendly Care Project there is...**^**b**^				
Higher job satisfaction	*n* = 24 3.88 (0.7)	*n* = 13 3.15 (1.1)	2.51	0.02
In general, how effective do you think the EFC Project have been in reducing … ..^**c**^				
Disrupted sleep	*n* = 26 3.81 (0.7)	*n* = 14 3.21 (0.9)	2.33	0.03
Episodes of delirium	*n* = 25 3.72 (0.6)	*n* = 14 3.21 (0.8)	2.21	0.03

^**a**^Likert scale was used to obtain helpfulness rating scores (1 = Not helpful to 5 = Extremely helpful)

^**b**^Likert scale was used to obtain agreement rating scores (1 = Strongly Disagree to 5 = Strongly Agree)

^**c**^Likert scale was used to obtain effectiveness rating scores (1 = Not effective to 5 Extremely Effective)

*P*-values are reported at 95% confidence level and calculated using Independent T-test for Equality of Means

Table 3 shows that the types of responses for accessing the Toolkit were significantly different between those who attended all 3 EFC LWs and those who attended less than 3 EFC workshops (*p* < 0.01). Participants that attended all 3 EFC LWs were significantly more likely to recommend the EFC Toolkit to others (87% versus 40%; χ^2^ = 8.82, *p* < 0.01) compared to those who attended less than 3 EFC LWs.

**Table 3:** Comparison between the number of workshops attended and utilizing EFC resources

Questions	Attended all 3 EFC learning workshops	Attended < 3 EFC learning workshops	χ^**2**^ Statistic	***p***-value
	n (%)	n (%)		
Have you accessed the online EFC Toolkit?				
Yes	10 (55.6%)	9 (22.5%)	9.69	< 0.01*
No	8 (44.4%)	18 (45.0%)		
I don’t Know	0 (0.00%)	13 (32.5%)		
Would you return and use the EFC Toolkit?				
Yes	13 (86.7%)	15 (48.4%)	3.71	0.05
No	0 (0.00%)	1 (3.2%)		
I don’t Know	2 (13.3%)	15 (48.4%)		
Would you recommend the EFC Toolkit to others?				
Yes	13 (86.7%)	12 (40.0%)	8.82**	< 0.01*
No	0 (0.00%)	0 (0.00%)		
I don’t Know	2 (13.3%)	18 (60.0%)		
Have you or your unit used EFC strategies to change your approach to caring for frail older patients				
Yes	17 (100%)	24 (77.4%)	0.71	0.40
No	0 (0.00%)	2 (6.5%)		
I don’t Know	0 (0.00%)	5 (16.1%)		

*Significant differences at 95% confidence level. **Pearson Chi-square test was used for comparison of this question as the ‘No’ responses were zero. For other questions, Chi-Square test for trend was used

There was no statistical difference in the scores between attending three workshops or less than three workshops related to EFC toolkit resources, practice change, site process, and outcomes.

Alongside the findings above, the data indicates that Toolkit resources were helpful to staff regardless of whether they had attended all 3 workshops, but that they were less likely to access these resources without doing so.

### Key informant interviews

Key Informant interviews continued to mirror and support earlier findings while adding more depth and nuance to them. EFC Change Team members felt LWs and resources were excellent, supported participant learning needs, and resulted in consistent practice change. They observed positive outcomes as a result of practice changes and education. Participants identified resource/staffing limitations and sustained buy-in as the most challenging. Further, their responses illustrated that sites/units consistently implemented changes that were best suited to and aligned with their own contexts, needs, challenges, and ongoing initiatives.

Three main themes emerged from interviews with EFC Change Team members;

- ➢ Positive Reactions to and Learning from the EFC workshops
- ➢ EFC Practice Change
- ➢ Project Outcomes and Patient Stories

#### Key reactions to and learning from the EFC workshops

The majority of participants reported an increase in their knowledge about caring for elderly patients. Most participants mentioned that they gained a better understanding of strategies to use when attending to elderly patients with dementia-related behaviours. Many were able to describe something specific they had learned (e.g. responsive behaviours, stages of dementia, or strategies and why they worked) and relate this knowledge to their patients. Most participants indicated that the LWs were very informative, relevant, and reinforced the whole concept of care and how to improve care for frail older adults:

- ➢ *“It helped to change my perspective in terms of how to nurse the elderly, looking deeper for what’s behind the behaviours that was probably the biggest thing I took from the workshops.”*
- ➢ *“I thought the ideas presented were really good, well presented, they were comprehensive*.”
- ➢ *“It gave us an opportunity to think differently, right, especially when they provided the interventions and the rationale behind it and the research showing us more driving force that there’s actually some split data that supports why.”*

In addition, LWs provided participants with practical information and knowledge on how to care for elderly patients suffering from dementia, delirium, etc., which increased their confidence and ability to incorporate EFC principles and strategies into their practices:

- ➢ *“I’m feeling like I’m starting to make a difference, understanding the dementia patient and digging deeper into the behaviours, is it medication, is it dehydration, is it lack of sleep and restlessness, and sometimes they just don’t sleep at night.”*

#### EFC practice change

Approaches to educating staff and family on units varied. Participants discussed how the EFC project team’s inclusion of Direct Care Staff in LWs supported implementation and practice change and highlighted their own approaches to educating staff and family afterwards.

- ➢ *“The involvement of the staff [at Workshops] to help get buy-in [was important]. You know as frontline workers with the patients on the unit it’s one thing for a manager to direct that, ‘we’re doing this project and here is what it’s all about’ but, when you get staff involved at the workshop, that helps make it happen on the unit.”*
- ➢ *“Our educator made posters and placed them around the unit and it was kind of word of mouth from staff that went and staff that didn’t. We had posters in patient rooms to get families involved. We had ‘5 Things You Know About Me’ posters in the room that families filled out so we knew more about the patients to ask them questions. We did more education and posters in the rooms about falls and why we don’t want to use medications. I think that’s the biggest.”*

Many participants emphasized PFCC as part of practice change, noting that family input was phenomenal when they were involved in the care of frail older adults. Staff who dialogued with patients’ families were able to obtain medical and social histories, and better understood patient behaviours. Most participants identified that educating the families about the patient worked best for family and staff health care relationship. They also felt that involving families requires a team effort to achieve the best care for patients:

- ➢ *“So that’s one change that we’ve done, we’ve tried to engage families right away. We try to ask them to fill out those social histories so that we get to know the patient and also the family’s able to contribute to the care planning. So they can tell us the best approach for the patient. Also, we invite them to family conferences very early, just because a transfer to psychiatric hospital, there’s a lot of stigma to that. So at least when you have them on board we talk to them, we tell them the plan, and they tell us their concerns and we work together as a team to meet those goals.”*

Participants devoted their time to the implementation of what they had learned from the Workshops, focusing on the following:

- Ambulation
  - ➢ *“We were looking at ambulation and how staying in bed and immobility actually contributes to the length of stay, it contributes to deconditioning and so we were currently getting patients walking sooner and in collaboration with a physio and then on that from the workshop we started monitoring how often our medicine patients were getting up in a chair for meals.”*
- Frail Scale and Restraints
  - ➢ *“We implemented the frail scale to determine the level of frailty of each of our admissions. We looked closer at our restraints methods, do we need to do physical restraint, and do we need chemical restraint. The behavioural tracking tool was a very important one, tracking behaviour and looking more at what might be behind behaviour and using physical and chemical restraints as a very last resort.”*
- Care Planning and Behaviour Mapping
  - ➢ *“ABC charting is actually a requirement now at our site for all admitted patients, they’re required to have 1 week to 14 days of the mapping and the ABC charting. And that really tremendously supports the care plan work and helps us identify those responsive behaviours and really why are they in hospitals and what do we need to work on. So that portion I would say is the biggest thing that is just completely embedded in our program now. And we also pull it forward again if we get a change in behaviours or change in presentation on any patient, tie that together with the PIECES assessment, tie in the behavioural mapping and PIECES worksheet for change in behaviour.”*
- Recreational Therapy and Meaningful Activities
  - ➢ *“Recreational therapy for our patients, so a program where we can have a little bit of recreational therapy in the evenings and weekend, we are implementing like games night, a movie night, hot drinks service for our elder friendly in the evenings. We’re looking at unit ambassadors for the elders.”*

#### Project outcomes

Direct Care Staff shared success stories about how patients’ care and outcomes had improved. In general, participants felt that there were declines in antipsychotic usage, falls, restraint use, and readmissions, as well as improvements in sleep and identification of behavioural pattern changes through behaviour mapping.

- ➢ *“I would think, anecdotally, there’s been some significant changes to the positive … I was just looking at survey results and complaints about care have gone from like 30% to 10%. I think they’re quarterly evaluations, 2 and half years ago it was 30% and now it’s 10%, and the predominant admission would be our geriatric frail elderly. So that would presume to me to say that the quality of care is improving. And it was a steady decline, it wasn’t like a spike here and there, it was a steady decline in the number of complaints.”*
- ➢ *We have a particular patient who was very, very aggressive and well his behaviour, he would hit, he would kick, he would speak, and you know, people were kind of scared to touch him. They would always give medications before even approaching him. And so since we have done the EFC project, I’ve kind of been educating them and saying to them ‘why didn’t you get to know this person more, why didn’t you approach him?’ People then understood that by just labeling him as aggressive, that already puts a blanket on him. So they focused more on approaching him rightly and being able to read and listen and watch out for his non-verbal [cues].*

Additionally, a number of participants reported that EFC had positively impacted their job satisfaction because they felt it helped them provide better care to their patients.

- ➢ *“Being part of EFC made me realize ‘okay, I am not the only one wanting the health system to move forward,’ there are also pockets of people out there who want the best for this patient, who want the best for the health system. So EFC has empowered me more, and made me more determined to make sure that people remember that the most important person in this whole thing is the patient. It’s not about budget, it’s not just about paperwork, it’s not just about trying to complete your day. It’s about that person who is in your care and leaving your work at the end of the day knowing that you have done everything you can for that person.”*

## Discussion

The pilot intervention of EFC practices in the acute care setting demonstrated successful implementation and positive benefits. The overall uptake of the program was lower than hoped in terms of the staff awareness and utilization of EFC resources and not all staff members were able to attend all learning workshops. However, participants who participated in learning workshops or accessed online resources were highly satisfied and demonstrated learning from them with consistent evidence of practice change. Further, participants identified a number of perceived positive outcomes from these changes.

### Learning workshops

Participants consistently reported increases in their awareness of, knowledge about, motivation to use, and subsequent uptake of EFC principles and practices, and linked these practice changes to EFC training and resources. Across sources, the data met the criteria for Levels 1 (positive reactions), 2 (learning), and 3 (practice change) of the Kirkpatrick Model; evidence also supported level 4 (outcomes). Further, participants consistently identified session strengths, including the content, format (e.g. case studies and group discussion), and facilitators. The learning workshop method of training and educating Direct Care Staff, such as nurses, was particularly important for achieving the desired practice changes [20]. The literature suggests that well educated Direct Care Staff in acute care areas can improve function, patient satisfaction and even reduce the risk of mortality in older people [20].

### Buy-in and resource use

The majority of participants had heard of EFC, supported the project and demonstrated high buy-in, and were aware of the primary principles and practice changes involved. Change Team members were more supportive of EFC principles and practices and accessed more resources.

In terms of the online EFC toolkit, both Change Team membership and LW attendance significantly impacted the likelihood that staff would access the Toolkit, though these factors did not impact helpfulness or satisfaction. The most important statistically significant impact of Workshop attendance was increased likelihood of accessing the EFC Toolkit, returning to it, and recommending it to others. The availability of the EFC toolkit to be accessed online through the internet portal was important as a medium for knowledge transfer [21]. Studies suggest that the healthcare staff usage of online resources is a significant proportion to the questions related to patient care; this might perhaps influence helpfulness or satisfaction [21].

Two challenges had the largest impacts on buy-in and resource use: access to the EFC Toolkit, and leadership/physician buy-in and support. Toolkit access included challenges around general awareness and the ability to locate this resource, and some participants were not able to access the Toolkit due to time constraints. Only 31% of survey participants received formal in-house training; further strategies to support awareness and access are required, particularly for Direct Care Staff not directly involved in site/unit implementation. Moreover, the Direct Care Staff/Workshop attendees consistently found leadership/physician buy-in problematic. Many participants, including the EFC project team, indicated that further targeting leadership/physician buy-in and education would be important to encourage more consistent implementation and outcomes. The leadership role ensures the safe delivery of quality care with modifiable factors for improvement [22]. Physician and Direct Care Staff buy-in is crucial as these individuals are intermediately framed in the intervention between patients and hospital settings [22].

### Practice change and perceived outcomes

Across data sources, participants reported practice changes at both the individual and site level. Participants provided insight and stories about how EFC had impacted their patients, site/unit, and themselves. In most cases, changes were positive, with an 85% rate of practice change reported in the Direct Care survey. EFC Change Team members, in particular, reported higher job satisfaction levels after EFC implementation at 8 EAS. Indeed, participants were especially happy to see practice changes and outcomes that primarily benefited patients and reflected PFCC. Finally, interviewees emphasized the ways in which they were able to make changes that suited their individual sites, needs, contexts, challenges, and ongoing initiatives. This flexibility, combined with the consistency of practice change, suggests that the project is well structured and flexible enough to build capacity for EFC across different contexts in Acute Care. These practice changes incorporate values, priorities, and goals of patients and families focusing on the fit of comprehensive care assessment and planning [23].

### Limitations

The compliance of frontline staff was not 100% with the EFC LWs. Frontline staff are very busy in their routine patient care work and had difficulty finding time for training workshops. Staff compliance with training has always been an issue, especially in very large organizations. In addition, the structural factors of the acute care context play a role in the implementation and uptake of such programs.

We used convenient sampling for the survey which was one of the limitations of this study as the sample included may not a fully representative sample of the population. However, this method provided easy accessibility and proximity to the Direct Care Staff for data collection purposes. The pilot implementation was geared toward determining feasibility for the implementation of EFC principles and practices. Since this was a pilot, system-level indicators such as decrease admissions and fewer pressure ulcers were not measured in this study. The larger Alberta-wide implementation of EFC will focus on these outcomes and will be reported in the future.

## Conclusions

The EFC approach is effective in creating positive reactions, learning, and practice changes at the pilot sites with sufficient buy-in, resources, education, and planning to support EFC principles and practices. This study recommends that the EFC pilot intervention can be adopted and implemented at a larger scale in all acute care settings to improve patient care. However, continued small, iterative changes are likely to benefit the project’s success and perhaps enhance the quality of care for frail older patients.

The future widespread implementation of the program needs to ensure

- Consistent engagement of stakeholders (e.g. educators, physicians, and leadership) who are well-situated to support Direct Care Staff are essential to the success of the project at a larger scale.
- Continue educational opportunities for Direct Care staff and family members, particularly in-person opportunities, and target education/resource supports for staff not directly involved in implementation (e.g. non-change team members, those not attending learning workshops).
- Alternative approaches such as mini-tutorials, case discussions in staff meeting need to consider to overcome the barrier of staff not accessing online resources
- Ensure there are clearly defined, accessible system-level indicators for evaluation and site-level data tracking to track the effectiveness of the program.

## Supplementary information

**Additional file 1.** Interview Guide. Brief description of the data: Interview Guide developed by authors for this study to conduct interviews with the key stakeholders.

**Additional file 2.** Comparison of participants reporting “Don’t Know” responses versus “other responses” for each question reported in Table 1. Brief description of the data: Some questions had a large number of “Don’t Know” response so this additional data provides the comparison between “Don’t Know” responses and “other responses”.

## Data Availability

The datasets generated and/or analyzed during the current study are not publicly available due organizational data sharing restrictions but are available from the corresponding author on reasonable request.

## Abbreviations

GDP: Gross Domestic Product
LOS: Length of Stay
EFC: Elder-Friendly Care
AHS: Alberta Health Services
SCN: Strategic Clinical Networks
ILC: Innovation Learning Collaborative
LW: Learning Workshop
EAS: Early Adoption Site
LPN: Licensed Practiced Nurse
RN: Registered Nurse
HCA: Healthcare Aide or Nursing Assistant
PFCC: Patient and Family Centered Care
CAM: Confusion Assessment Method
ABC Charting: Antecedent-Behaviour Consequence Charting
PIECES: Physical Intellectual Emotional Capabilities Environment Social

## Footnotes

1 AHS is a fully-integrated single health authority for the Canadian province of Alberta delivering services to more than four million residents. More information: https://www.albertahealthservices.ca/about/about.aspx

2 SCNs are embedded within AHS to act as change agents driving and inspiring health innovation to improve patient outcomes. Seniors Health SCN strives to optimize senior health, well-being and independence through evidence-based best practices.

3 The EFC project was designed to align with several new and ongoing initiatives in AHS, including a new Restraint as a Last Resort Policy and an ongoing collaborative care initiative (CoAct). Our analysis indicated that alignment with these initiatives contributed to pilot successes.

4 EFC resources identify a number of frailty risks and characteristics, including cognitive challenges, falls, functional dependence, 3–5 + medications, 2 or more medical conditions, slow gait speed, lack of social support, weight loss, and exhaustion.

## References

[1] Chiou S, Chen L (2009). Towards age-friendly hospitals and health services. Arch Gerontol Geriatr.

[2] Statistics Canada (2011). Census of population.

[3] Huang A, Larente N, Morais J. Moving towards the Age-friendly hospital. A paradigm shift for the hospital-based care of the elderly. Can Geriatr J. 2011;14(4):100–3. 10.57700/cgj.v14i4.8. 10.57700/cgj.v14i4.8 PMC3516236 23251321

[4] Canadian Institute for Health Information (CIHI) (2018). Health Spending.

[5] Mate K, Berman A, Laderman M, Kabcenell A, Fulmer T (2018). Creating age-friendly health systems – a vision for better care of older adults. Healthcare.

[6] Surkan M, Gibson W (2018). Interventions to mobilize elderly patients and reduce length of hospital stay. Can J Cardiol.

[7] Collamati A, Martone A, Poscia A, Brandi V, Celi M, Marzetti E (2015). Anticholinergic drugs and negative outcomes in the older population: from biological plausibility to clinical evidence. Aging Clin Exp Res.

[8] Wimmer B, Dent E, Bell J, Wiese M, Chapman I, Johnell K (2014). Medication regimen complexity and unplanned hospital readmissions in older people. Ann Pharmacother.

[9] Hubbard R, O’Mahony M, Woodhouse K (2012). Medication prescribing in frail older people. Eur J Clin Pharmacol.

[10] Inouye S (2006). Delirium in older persons. N Engl J Med.

[11] Verma J, O'Connor P, Hodge J, Abrams H, Bennett J, Sinha S (2017). Healthcare for the aging citizen and the aging citizen for healthcare: involving patient advisors in elder-friendly care improvement. Healthcare Quarterly.

[12] Gordon A, Masud T, Gladman J (2013). Now that we have a definition for physical frailty, what shape should frailty medicine take?. Age Ageing.

[13] Wong KS, Ryan DP, Liu BA (2014). A system-wide analysis using a senior-friendly hospital framework identifies current practices and opportunities for improvement in the care of hospitalized older adults. J Am Geriatr Soc.

[14] Pearce S, Rogers-Clark C, Doolan J (2011). A comprehensive systematic review of age-friendly nursing interventions in the management of older people in emergency departments. JBI Database System Rev Implement Rep.

[15] Hart B, Frank C, Hoffman J, Dickey D, Kristjansson J (2006). Senior friendly health services. Perspectives.

[16] Fulmer T, Li N (2018). Age-friendly health Systems for Older Adults with Dementia. J Nurse Pract.

[17] Galvin J, Kuntemeier B, Al-Hammadi N, Germino J, Murphy-White M, McGillick J (2010). “Dementia-friendly hospitals: care not crisis”—improving the care of the hospitalized patient with dementia. Alzheimers Dement.

[18] Thompson C, Aitken L, Doran D, Dowding D (2013). An agenda for clinical decision making and judgement in nursing research and education. Int J Nurs Stud.

[19] Thomas D (2006). A general inductive approach for analyzing qualitative evaluation data. Am J Eval.

[20] Coster S, Watkins M, Norman I (2018). What is the impact of professional nursing on patients’ outcomes globally? An overview of research evidence. Int J Nurs Stud.

[21] Curran VR, Fleet L (2005). A review of evaluation outcomes of web-based continuing medical education. Med Educ.

[22] Parke B, Hunter K, Bostrom A, Chambers T, Manraj C (2012). Identifying modifiable factors to improve quality for older adults in hospital: a scoping review. Int J Older People Nursing.

[23] Pelton L, Fulmer T, Hendrich A, Mate K (2017). Creating age-friendly health systems. Healthc Exec.

