# Supplementary material for: Pilot implementation of elder-friendly care practices in acute care setting: a mixed methods study
Source: BMC Health Serv Res. 2020 Apr 24;20:347. doi: 10.1186/s12913-020-05091-y (PMC7181575; doi:10.1186/s12913-020-05091-y)
Supplement: Supplementary file 1 — Additional file 1. Interview Guide. Brief description of the data: Interview Guide developed by authors for this study to conduct interviews with the key stakeholders. [file 12913_2020_5091_MOESM1_ESM.docx]

**Participant Interview Guide**

First I would like to talk a bit about you and your role in Elder Friendly Care (EFC)

1. What is your role in the EFC project?
   1. How long have you been working on this project?
   2. How did you become involved?
   3. What attracted you to this work?
   4. How has your role changed over time?

Next, I would like to talk about how EFC was rolled out in the pilot acute sites:

1. How did your team go about developing EFC resources and education?

(probes: challenges, successes,)

1. What was your team’s approach to rolling out EFC in Acute care?
   1. How did previous Appropriate Use of Antipsychotic (AUA) work inform this project?
   2. How did your teams’ approach for EFC in acute care?
   3. How did you recruit pilot sites to participate in EFC?
   4. How did you engage with sites throughout the pilot

(probes: initial engagement, on-going support, workshops, action periods, leaders’ orientations)

- 1. How did you incorporate site feedback into the project? What changes did you make to the project and/or provincial spread plans based on this feedback?

1. What resources did you find most useful during this project?
   1. Resources used by the EFC team for developing and delivering EFC education & support?
   2. Resources used by the EFC team for supporting sites during roll out?
   3. EFC resources that sites found useful?
2. What was most challenging for your team in terms of developing and implementing EFC? (Probes: resources, staffing, funding, etc.)
3. What was the most successful part of developing and implementing EFC?
4. How engaged did you feel acute sites were throughout this process?
   1. What worked best for engaging sites? (probe: facilitators)
   2. What was most challenging about engaging sites? (probe: barriers)
   3. Did you notice any differences in engagement between continuing care and acute care?
5. Can you tell me some of the success stories you heard from acute sites?
6. Can you tell me about the kinds of challenges sites shared with you throughout the roll-out?
   1. How did you support sites in addressing these challenges?
   2. Were any sites unable to address and/or resolve challenges? Why do you think that is?

Finally, I would like look forward to the future of EFC.

1. Based on your experiences during implementation, do you think that acute sites will be able to sustain EFC? Why or why not?
   - 1. What do you think are the necessary ingredients for sustaining EFC?

(Probes: AHS resources for supporting sites, Site resources, buy-in, etc.)

Thank you for your time!
