# Supplementary material for: Pilot implementation of elder-friendly care practices in acute care setting: a mixed methods study
Source: BMC Health Serv Res. 2020 Apr 24;20:347. doi: 10.1186/s12913-020-05091-y (PMC7181575; doi:10.1186/s12913-020-05091-y)
Supplement: Supplementary file 2 — Additional file 2. Comparison of participants reporting “Don’t Know” responses versus “other responses” for each question reported in Table 1. Brief description of the data: Some questions had a large number of “Don’t Know” response so this additional data provides the comparison between “Don’t Know” responses and “other responses”. [file 12913_2020_5091_MOESM2_ESM.docx]

| **Additional Table 1A**  Comparison of participants reporting Don’t Know responses versus other responses for each question reported in Table 1. | | | | |
| --- | --- | --- | --- | --- |
|  | Don’t Know Response | Other Responses (Yes/No) | χ^2^ Statistic | p-value |
| *Have you accessed the online EFC Toolkit?* | | | | |
| Management staff (n=8)  LPN (n=15)  RN (n=17)  HCA (n=5)  Unit Clerk (n=2)  Others (n=11) | 0%  33.3%  17.6%  0.0%  50.0%  36.4% | 100%  66.7%  82.4%  100.0%  50.0%  63.6% | 0.29 | 0.58 |
| *Would you return and use the EFC Toolkit?* | | | | |
| Management staff (n=8)  LPN (n=11)  RN (n=13)  HCA (n=5)  Unit Clerk (n=1)  Others (n=8) | 37.5%  36.4%  15.4%  40.0%  100.0%  62.5% | 62.5%  63.6%  84.6%  60.0%  0.0%  37.5% | 1.72 | 0.19 |
| *Would you recommend the EFC Toolkit to others?* | | | | |
| Management staff (n=8)  LPN (n=11)  RN (n=12)  HCA (n=5)  Unit Clerk (n=1)  Others (n=8) | 37.5%  54.5%  33.3%  20.0%  100.0%  62.5% | 62.5%  45.5%  66.7%  80.0%  0.0%  37.5% | 0.06 | 0.79 |
| *Have you or your unit used EFC strategies to change your approach to caring for frail older patients* | | | | |
| Management staff (n=8)  LPN (n=11)  RN (n=14)  HCA (n=5)  Unit Clerk (n=1)  Others (n=9) | 0.0%  9.1%  7.1%  20.0%  0.0%  22.2% | 100.0%  90.9%  92.9%  80.0%  100.0%  77.8% | 0.01 | 0.92 |
| **Significant differences at 95% confidence level* | | | | |
